# Supplementary material for: Identification of Freezing-Responsive microRNAs and Their Targets in Chinese Jujube by Small RNA and Degradome Sequencing
Source: Int J Mol Sci. 2026 Sep 4;27(17):7912. doi: 10.3390/ijms27177912 (PMC13566890; doi:10.3390/ijms27177912)
Supplement: Supplementary file 1 [file ijms-27-07912-s001.zip › Supplementary Material S2/novel_miR_9.pdf]

Provisional ID : novel_miR_9
Score total : 3.6
Score for star read(s) : -1.3
Score for read counts : 0
Score for mfe : 3.3
Score for randfold : 1.6
Score for cons. seed :
Total read count : 2198
Mature read count : 1460
Loop read count : 722
Star read count : 16

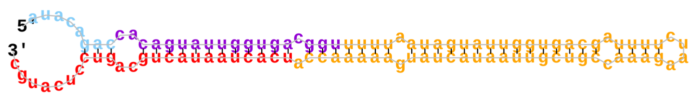

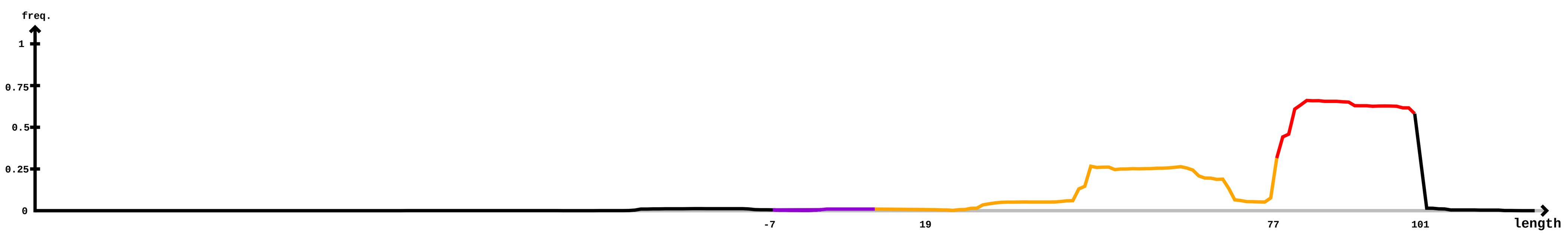

Star Mature

```
5'- augugaucauaguaaauaaggguucagauacauugugaucauaguaaauuagguuuuagauguauauauauauauauauauauauauauauaucaguccguuuauagugcggauggucuucauacagaccacaguauuggugacgguuuuuaaauaguauuggugacgauuuucuaagaaaccgucguuaaauacuaugaaaaaccaucacuaaauacugcaguccucaugcggaccguauucacuauagaau -3'  obs
    augugaucauaguaaauaaggguucagauacauugugaucauaguaaauuagguuuuagauguauauauauauauauauauauauauauauaucaguccguuuauagugcggauggucuucauacagaccacaguauuggugacgguuuuuaaauaguauuggugacgauuuucuaagaaaccgucguuaaauacuaugaaaaaccaucacuaaauacugcaguccucaugcggaccguauucacuauagaau       exp
    .((((((((((((..................))))))))))))........((.....((((((((((((((((....))))))))))))))))...)).(((((((((...(((((((.(((...(((..((((((((((((.(((((((.((((((((((((((((.((((....)))).)))))))))))))))).))))))).))))))))))))..)))...))).)))))))...)))))))))..  reads  mm  sample
    ............................................................uguauCuauauauauauauauauaua.......................................................................................................................................................................  1  1  LCK-3
    ............................................................................................uGaguccguuuauagugc...............................................................................................................................................  1  1  LT-1
    .................................................................................................Ucguuuauagugcggaugguc.......................................................................................................................................  1  1  LT-1
    ..................................................................................................cguCuauagugcggauggu........................................................................................................................................  5  1  LT-2
    ..................................................................................................cguuuauagugcggauggucC......................................................................................................................................  1  1  JCK-3
    ...................................................................................................guuuauagugcggaugguc.......................................................................................................................................  3  0  LT-2
    ...................................................................................................guuuauagugcggaugguc.......................................................................................................................................  1  0  JCK-1
    ...................................................................................................guGuauagugcggaugguc.......................................................................................................................................  1  1  LT-3
    ...................................................................................................guuuauagugcggaugguc.......................................................................................................................................  1  0  JCK-2
    ...................................................................................................guuuauagugcggaugguc.......................................................................................................................................  2  0  LT-1
    ...................................................................................................guCuauagugcggauggucu......................................................................................................................................  1  1  LT-1
    ...................................................................................................guuuauGgugcggauggucuuc....................................................................................................................................  1  1  LT-2
    ...................................................................................................guuuauagugcggauggucuAc....................................................................................................................................  1  1  LT-2
    ...................................................................................................guCuauagugcggauggucuuca...................................................................................................................................  3  1  LT-3
    ...................................................................................................guCuauagugcggauggucuuca...................................................................................................................................  1  1  LT-1
    .....................................................................................................uuauagugcggauggucCu.....................................................................................................................................  2  1  LT-2
    .......................................................................................................auagugcggauggucuuca...................................................................................................................................  1  0  JCK-3
    .......................................................................................................auagugcggauggucuucauG.................................................................................................................................  1  1  JCK-3
    ...........................................................................................................ugcggauggucCucauacaga.............................................................................................................................  1  1  LT-3
    ............................................................................................................gcggauggucuuUauaca...............................................................................................................................  1  1  JCK-2
    .....................................................................................................................cuucauGcagaccacaguauugg.................................................................................................................  1  1  LCK-1
    ........................................................................................................................cauacagacUacaguauugg.................................................................................................................  2  1  LT-3
    ................................................................................................................................ccacaguauuggugaAgguu.........................................................................................................  1  1  JCK-2
    ................................................................................................................................ccacaguauuggugacgguuuuu......................................................................................................  3  0  JT-3
    .................................................................................................................................cacaguauuggugacggu..........................................................................................................  2  0  JCK-1
    .................................................................................................................................cacaguauuggugacggu..........................................................................................................  5  0  LT-3
    ..................................................................................................................................acaguauuggugacgguu.........................................................................................................  1  0  LT-3
    ..................................................................................................................................acaguauuggugacgguu.........................................................................................................  1  0  LT-1
    ..................................................................................................................................acaguauuggugacgguuu........................................................................................................  1  0  LT-1
    ..................................................................................................................................acaguGuuggugacgguuu........................................................................................................  2  1  LT-3
    ..................................................................................................................................acaguauuggugacgguuCu.......................................................................................................  1  1  LT-3
    ..................................................................................................................................acaguauuggugacgguuuuu......................................................................................................  1  0  JCK-3
    ..................................................................................................................................acaguauuggugacgguuuuu......................................................................................................  1  0  JCK-2
```

Star · **Mature**

augugaucauaguaaauaaggguucagauacauugugaucauaguaaauuagguuuuagauguauauauauauauauauauauauauauauaucaguccguuuauagugcggauggucuucauacagaccacaguauuggugacgguuuuuaauaguauuggugacgauuuucuaagaaaccgucguuaauacuaugaaaaaccaucacuaauacugcaguccucaugcggaccguauucacuauagaau

acaguauuggugacgguuuuu	1	0	LT-1
gacgguuuuuGauaguauu	1	1	LT-2
cgguuuuuaauaguauuggug	1	0	JCK-2
uuuuaauaguauuggugaUgauuuu	1	1	JCK-1
uuuuaauaguauuggugaUgauuuu	1	1	JCK-3
auaguauuggugacgauu	1	0	LT-1
auaguaCuggugacgauuu	1	1	JCK-3
auaguauuggugacgauuuu	1	0	JCK-3
auaguauuggugacgauuuuc	1	0	JCK-1
auaguauuggugacgauuuucu	1	0	JCK-2
auaguauuggugacgauuuucu	1	0	LT-1
auaguauuggugacgauuuucuG	3	1	LT-2
auaguauuggugGcgauuuucuaa	1	1	JCK-2
uaguaCuggugacgauuuucuaaga	2	1	JCK-2
aguGuuggugacgauuuucu	1	1	LT-1
aguGuuggugacgauuuucuaaga	1	1	JCK-1
aguauuggugacgauuuucuaaga	2	0	JCK-2
aguauuggugacgauuuucuaaga	4	0	LCK-3
aguGuuggugacgauuuucuaaga	3	1	JCK-2
aguauuggugacgauuuucuaaga	2	0	JCK-1
aguGuuggugacgauuuucuaaga	1	1	LT-1
aguauuggugacgauuuucuaaga	3	0	JCK-3
guauuggugacgauuuucuGa	1	1	JCK-2
guauuggugacgauuuucuGa	1	1	JCK-1
uaAuggugacgauuuucua	17	1	LCK-1
uaAuggugacgauuuucua	21	1	LCK-2
uauuggugacgauuuucuaaga	1	0	LT-3
uaCuggugacgauuuucuaaga	1	1	JCK-3
uaCuggugacgauuuucuaaga	2	1	JCK-1
uaCuggugacgauuuucuaaga	2	1	JCK-2
uGuuggugacgauuuucuaagaa	2	1	LT-3
auuCgugacgauuuucuaaga	2	1	LT-2
auuggugacgauuuucuaaga	1	0	LT-2
aCuggugacgauuuucuaagaa	1	1	LT-1
auuggugacgauuuucuaagaaac	3	0	JCK-2
auuggugacgauuuucuaagaaac	4	0	JCK-1
auuggugacgauuuucuaagaaac	4	0	JCK-3
uuggugacgauuuucuaaga	1	0	LT-3
uuggugacgauuuucuaaga	1	0	JCK-2
uuggugacgauuuucuaaga	3	0	JT-1
uuggugacgauuuucuaagaa	1	0	LT-2
Cuggugacgauuuucuaagaa	1	1	JCK-2
uuggugacgauuuucuaagaaU	2	1	LT-1
uuggugacgauuuucuaagaUa	3	1	LT-2
uggugCcgauuuucuaaga	1	1	LT-1
uggugacgauuuucuaaga	2	0	LT-2
uggugacgauuuucuaaga	3	0	JCK-2
uggugacgauuuucuaaga	1	0	LT-3
uggugacgauuuucuaagaa	1	0	LT-3
uggugacgauuuucuaagaU	1	1	LT-3
ggugacgauuuucuaaga	4	0	LT-3
ugacgauuuGcuaagaaacc	1	1	LT-3
gacgauuuucuaagaaacc	1	0	LT-2
uuuucuaagaaaccgucguu	1	0	LT-1
uuucuaagaaaccgucguu	2	0	LT-3
uuucuaagaaaccgucguuaa	1	0	JCK-3
uuucuaagaaaccgucguuaaua	1	0	LT-1
uuucuaagaaaccgucguuaaua	1	0	JCK-2
uuucuaagaaaccgucguuaauac	1	0	JCK-2
uuucuaagaaaccgucguuGauac	1	1	LT-3
uucuaagGaaccgucguuaau	1	1	LT-3
uucuaagaaaccgucguuaaua	1	0	LT-1
uucuaagaaaccgucguuaaua	1	0	JCK-2
uucuaagaaaccgucguuaaua	1	0	JCK-3
uucuaagaaaccgucguuaauacu	1	0	LT-3
uucuaagaaaccgucguuaauacu	1	0	JCK-2
uucuaagaaaccgucguuaauacu	2	0	JCK-3
uucuaagaaaccgucguuaauacu	1	0	JCK-1
uucuaagaaaccgucguuaauacu	1	0	LT-1
ucuaagaaaccgucguuaaua	2	0	LT-1

Star Mature

augugaucauaguaaauaaggguucagauacauugugaucauaguaaauuagguuuuagauguauauauauauauauauauauauauauauaucaguccguuuauagugcggauggucuucauacagaccacaguauuggugacgguuuuuaauaguauuggugacgauuuucuaagaaaccgucguuaauacuaugaaaaaccaucacuaauacugcaguccucaugcggaccguauucacuauagaau

ucuaagaaaccgucguuaauacA	1	1	LT-1
cuaagaaaccgucguuaa	2	0	LT-1
cuaagaaaccgucguuCa	1	1	LT-3
cuaagaaaccgucguuaC	2	1	LT-2
cuaagaaaccgucguuaa	8	0	LT-2
cuaagaaaccgucguuaa	3	0	LT-3
cuaagGaaccgucguuaa	2	1	LT-2
cuaagaaaccgucguuaau	3	0	JCK-1
cuaagaaaccgucguuaau	4	0	JCK-2
cuaagaaaccgucguuaau	1	0	JCK-3
cuaagaaaccgucguuaau	12	0	LCK-3
cuaagaaaccgucguuaau	1	0	LT-3
cuaagaaaccgucguuaau	6	0	LT-2
cuaagaaaccgucguuaauU	3	1	JCK-2
cuaagaaaccgucguuGaua	3	1	LT-2
cuaagaaaccgucguuaaua	4	0	LT-1
cuaagaaaccgucguuaauU	9	1	LT-3
cuaagaaaccgucguuaauU	2	1	LT-1
cuaagaaaccgucguuaauU	1	1	JCK-1
cuaagaaaccgucguuaaua	4	0	JCK-3
cuaagaaaccgucguuaaua	2	0	LT-2
cuaagaaaccgucguuaauU	3	1	LCK-3
cuaagaaaccgucguuaauU	4	1	JCK-3
cuaagaaaccgucguuaaua	3	0	JCK-1
cuaagaaaccgucguuaaua	10	0	LCK-3
cuaagaaaccgucguuaauC	1	1	JCK-1
cuaagaaaccgucguuaauU	11	1	LT-2
cuaagaaaccgucguuaaua	1	0	JCK-2
cuaagaaaccgucguuaaua	6	0	LT-3
cuaagaaaccgucguuaauac	4	0	LT-3
cuaagaaaccgucguuaauac	2	0	LT-2
cuaagaaaccgucguuaauac	3	0	JCK-1
cuaagaaaccgucguuaauac	14	0	LCK-3
cuaagaaaccgucguuCauac	1	1	LT-3
cuaagaaaccgucguuaauac	3	0	JCK-2
cuaagaaaccgucguuaauacu	1	0	LT-1
cuaagaaaccgucguuaauacu	6	0	LT-2
cuaagaaaccgucguuGauacu	1	1	LT-3
cuaagaaaccgucguuaauacu	1	0	LT-3
cuaagaaaccgucguuaauacu	3	0	JCK-3
cuaagaaaccgucguuaauacu	2	0	JCK-2
cuaagaaaccgucguuaauacua	3	0	LT-2
cuaagaaUccgucguuaauacua	1	1	JCK-1
cuaagaaaccgucguuaauacuU	1	1	LT-3
cuaagaaaccgucguuaauacuau	1	0	JCK-3
cuaagaaaccgucguuaauacuau	4	0	JCK-2
cuaagaaaccgucguuaauacuau	5	0	JCK-1
uaagaaaccgucguuaaua	2	0	LT-3
uaagaaaccgucguuaauU	1	1	LT-3
uaagaaaccgucguuaaua	1	0	LT-1
uaagaaaccgucguuaauac	1	0	LT-1
uaagaaaccgucguuaauacua	1	0	LT-3
uaaNaaaccgucguuaauacuaug	1	1	JCK-1
uaagaaaccgucguuaauacuaug	7	0	JCK-3
uaagaaaccgucguuaauacuaug	1	0	LT-1
uaagaaaccgucguuaauacuaug	11	0	JCK-2
uaagaaCccgucguuaauacuaug	1	1	JCK-3
uaagaaaccgucguuaauacuauU	1	1	LT-1
uaagaaaccgucguuaauacuaug	1	0	LT-3
uaagaaaccgucguuaauacuaug	9	0	JCK-1
aagaaaccgucguuaaua	3	0	JCK-2
aagaaaccgucguuaaua	1	0	LT-3
aagaaaccgucguuaauacu	2	0	LT-2
aagaaaccgucguuaauacG	1	1	LT-1
aagaaCccgucguuaauacu	1	1	LT-2
aagaaaccgucguuaauacu	5	0	LT-3
aagaaaccgucguuaauacu	1	0	JCK-1
aagaaaccgucguuaauacu	1	0	JCK-2
aagaaaccgucguuaauacu	3	0	JCK-3
aagaaaccgucguuaauacA	6	1	LT-2

Star Mature

augugaucauaguaaauaaggguucagauacauugugaucauaguaaauuagguuuuagauguauauauauauauauauauauauauauaucaguccguuuauagugcggauggucuucauacagaccacaguauuggugacgguuuuuaauaguauuggugacgauuuucuaagaaaccgucguuaauacuaugaaaaaccaucacuaauacugcaguccucaugcggaccguauucacuauagaau

aagaaaccgucguuaauaGu	2	1	LT-2
aagaaaccgucguuaauacua	1	0	LT-3
aagaaaccgucguuaauaGua	2	1	JCK-1
aagaaaccgucguuaauacua	3	0	JCK-3
aagaaaccgucguuaauacuU	2	1	LT-3
aagaaaccgucguuGauacuau	1	1	JCK-1
aagaaaccgucguuaauacuau	2	0	JCK-2
aagaaaccgucguuaauacuaug	4	0	LT-1
aaUaaaccgucguuaauacuaug	1	1	JCK-2
aagGaaccgucguuaauacuaug	3	1	JCK-3
aagGaaccgucguuaauacuaug	1	1	JCK-2
aagGaaccgucguuaauacuaug	1	1	LT-3
aagaaaccgucguuaauacuaug	10	0	LT-3
aaUaaaccgucguuaauacuaug	5	1	JT-2
aagaaaccgucguuaauacuaug	19	0	JCK-2
aagaaaccgucguuaauacuaug	24	0	JCK-1
aagaaaccgucguuaauacuaug	17	0	JCK-3
aaUaaaccgucguuaauacuaug	1	1	LT-1
aagaaaccgucguuCauacuauga	5	1	JCK-3
aaUaaaccgucguuaauacuauga	4	1	JCK-2
aaUaaaccgucguuaauacuauga	6	1	JCK-3
aagaaaccgucguuCauacuauga	1	1	JCK-1
aagaaaccgucguuaauacuauga	46	0	JCK-2
aagaaaccgucguuaauacuauga	27	0	JCK-1
aagGaaccgucguuaauacuauga	1	1	JCK-2
aagaaaccgucguuaauacuauga	6	0	LT-1
aagaaaccgucguuCauacuauga	2	1	LT-2
aagaaaccgucguuaauacuauga	6	0	LT-2
aagaaaccgucguuaauacuauga	29	0	JCK-3
aagaaaccgucguuCauacuauga	3	1	JCK-2
aaUaaaccgucguuaauacuauga	1	1	LT-1
aagaCaccgucguuaauacuauga	1	1	JCK-2
aagaaaccgucUuuaauacuauga	1	1	JCK-2
aagaaaccgucguuaauaGuauga	1	1	LT-1
aaUaaaccgucguuaauacuauga	3	1	JCK-1
aagaaaccgucUuuaauacuauga	1	1	JCK-3
aagaaaccgucUuuaauacuauga	7	1	LCK-3
aagaaaccgucguuaauacuauga	2	0	LT-3
aagaaaccgucUuuaauacuaugaa	3	1	JCK-2
aagaaaccgucguuaauacuaugaa	1	0	LT-1
aagaaaccgucUuuaauacuaugaa	1	1	JCK-1
aagaaaccgucUuuaauacuaugaa	2	1	JCK-3
aagaaaccgucguuaauacuaugaU	1	1	LT-1
agaaaccgucguuaauacua	4	0	JCK-3
agaaaccgucguuaauacuaug	3	0	LT-1
agaaaccgucguuaauacuaug	2	0	LT-3
agaaaccgucguuaauacuaug	1	0	JCK-1
agaaaccgucguuaauacuaug	2	0	JCK-3
agaaaccgucguuaauacuauC	2	1	LT-1
agaaaccgucguuaauacuauga	1	0	JCK-3
agaaaccgucguuaauacuauga	1	0	JCK-2
agaaaccgucguuCauacuauga	2	1	JCK-3
agaaaccgucguuCauacuauga	2	1	JCK-2
agaaaccgucUuuaauacuauga	4	1	LCK-3
gaaaccAucguuaauacuaug	1	1	LT-3
gaaaccAucguuaauacuaug	2	1	JCK-3
gaaaccgucguuaauacuaug	1	0	LT-3
gaaaccAucguuaauacuaug	1	1	JCK-2
gaaaccgucguuCauacuaugaa	1	1	JCK-1
gaaaccgucguuaauaAuaugaa	1	1	LT-2
aaaccgucUuuaauacuaugaaa	1	1	JCK-1
aaccgucguuaauacuau	1	0	JCK-3
aacGgucguuaauacuaug	1	1	LT-3
aaccgucguuaauacuaug	1	0	LT-1
aaccgucguuaauacuauga	1	0	LT-3
aaccgucUuuaauacuaugaaaa	1	1	LT-2
aaccgucguuaauacuaugaaaaa	1	0	LT-3
aacGgucguuaauacuaugaaaaa	1	1	JCK-2
aacGgucguuaauacuaugaaaaa	1	1	JCK-3
accgucguuaauacuaugaaaaa	1	0	JCK-3

Star **Mature**

augugaucauaguaaauaaggguucagauacauugugaucauaguaaauuagguuuuagauguauauauauauauauauauauauauauaucaguccguuuauagugcggauggucuucauacagaccacaguauuggugacgguuuuuaauaguauuggugacgauuuucuaagaaaccgucguuaauacuaugaaaaaccaucacuaauacugcaguccucaugcggaccguauucacuauagaau

accgucguuaauacuaugaaaaa	2	0	JCK-2
accgucguuaauacuaugaaaaac	2	0	JCK-3
accgucguuaauacuaugaaaaac	3	0	JCK-1
accgucguuaauacuaugaaaaac	1	0	LT-1
accgucguuaauacuaugaaaaaU	1	1	LT-3
Gccgucguuaauacuaugaaaaac	1	1	JCK-1
accgucguuaauacuaugaaaaac	4	0	JCK-2
ccgucguuaauacuauga	1	0	LT-3
ccgucguuaauacuaugaaaaacc	5	0	JCK-3
cgucguuaauacuaugaaa	15	0	JT-1
ucguuCauacuaugaaaaaccauc	2	1	JCK-3
cguuaauacuaugaaaaaccauca	1	0	LT-2
guuaauacuaugaaaaaccauca	1	0	JCK-1
guuaauacuaugaaaaaccauca	2	0	JCK-2
guuaauacuaugaaaaaccauca	2	0	JCK-3
uuaauacuaugaaaaaccaucacu	1	0	LT-1
uaauacuaugaaaaaccaucacua	3	0	JCK-1
uaauacuaugaaaaaccaucacuC	1	1	LT-3
Cauacuaugaaaaaccauc	2	1	LT-2
aauacuCugaaaaaccauc	1	1	LT-3
aauacuaugaaaaaccauca	1	0	LT-2
aauacuaugaaaaaccaucacua	1	0	JCK-2
aauacuaugaaaaaccaucacua	1	0	LT-3
aauacuaugaaaaacUaucacua	1	1	LT-1
aauacuaugaaaaaccaucacua	1	0	JCK-3
aauacuaugaaaaaccaucacua	1	0	LT-1
aauacuaugaaaaaccaucacua	2	0	JCK-1
auacuaugaaaaaccaucacuaau	2	0	JCK-3
auacuaugaaaaaccaucacuaau	1	0	LT-1
auacuCugaaaaaccaucacuaau	2	1	JCK-2
aGacuaugaaaaaccaucacuaau	2	1	JCK-2
aGacuaugaaaaaccaucacuaau	1	1	JCK-3
auacuaugaaaaaccaucacuaau	1	0	JCK-2
augaaaaaccaucacuaCuacug	2	1	JCK-1
augaaaaaccaucacuaauacugc	2	0	LT-3
augaaaaacUaucacuaauacugc	1	1	LT-2
augaaaaaccaucacuaauacugU	3	1	JCK-3
augaaaaaccaucacuaauacugc	10	0	JCK-2
augaaaaaccaucacuaauacugc	4	0	LCK-3
augaaaaaccaucacuaauacugc	4	0	JCK-1
augaaaaacUaucacuaauacugc	3	1	JCK-2
augaaaaaccaucacuaauaUugc	1	1	LT-1
augaaaaaccaucCcuaauacugc	1	1	JCK-2
augaaaaaccaucacuaauacugU	1	1	JCK-2
augaaaaaccaucacuaauacugc	2	0	LT-1
augaaCaaccaucacuaauacugc	1	1	JCK-3
augaaaaaccaucacuCauacugc	1	1	JCK-2
augaaaaaccaucacuaauacugc	3	0	JCK-3
Cugaaaaaccaucacuaauacugc	2	1	LT-2
ugaaaaaccaucacuaauacu	1	0	LT-1
gaaaaaccaucacuaauacu	3	0	LT-2
gaaaaaccaucacuaauacu	1	0	LT-1
gaaaaaccaucacuaauacu	1	0	LT-3
gaaaaaccaucacuaauacug	1	0	JCK-2
gaaaaaccaucacuaCuacug	1	1	LT-1
gaaaaaccaucacuaauacug	1	0	JCK-3
gaaaaaccaucacuaauacugc	1	0	LT-3
gaaaaaccaucacuaauacugc	2	0	JCK-1
gaaaaaccaucacuaCuacugc	2	1	JCK-1
gaaaaaccaucacuaCuacugc	1	1	JCK-2
gaaaaaccaucacuaauaUugca	1	1	LT-1
gaaaaacUaucacuaauacugcag	2	1	JCK-3
aaaaaccaucacuaauacugc	1	0	JCK-1
aaaaaccaucacuaauacugc	1	0	JCK-3
aaaaaccaucacuaauacugcagu	2	0	JCK-2
aaaaaccaucacuaauacugcagu	1	0	JCK-3
aaaaccaucacuaauacugc	2	0	LT-3
aaaaccaucacuaauacugc	1	0	LT-1
aaaaccaucacuaauacugcagu	1	0	JCK-2
aaaaccaucacuaauacugcagu	1	0	JCK-3

Star / **Mature**

augugaucauaguaaauaaggguucagauacauugugaucauaguaaauuagguuuuagauguauauauauauauauauauauauauauauaucaguccguuuauagugcggauggucuucauacagac**cacaguauuggugacggu**uuuuaauaguauuggugacgauuuucuaagaaaccgucguuaaauacuaugaaaaacca**ucacuaauacugcaguccucaugc**ggaccguauucacuauagaau

...aaccaucacuaauacugcaguAcu...	1	1	JCK-1
...ccaucacuaauacugcagA...	1	1	LT-3
...ccaucacuaauacugcaguccuc...	2	0	LT-2
...caucacuaauacugcagu...	1	0	JCK-2
...caucacuaauacugcaguAcu...	2	1	LT-3
...Uaucacuaauacugcaguccuc...	1	1	JCK-1
...caucacuaauacugcaguGcuca...	1	1	LT-1
...caucacuaauacugcaguccuca...	1	0	LT-1
...caucaUuaauacugcaguccuca...	2	1	JCK-1
...caucaUuaauacugcaguccuca...	1	1	JCK-3
...caucacuaauacugcaguccucau...	1	0	JCK-2
...aucacuaauacugcaguccu...	1	0	LT-1
...aucacuaauacugUaguccuca...	4	1	JCK-2
...aucacuaauacugcaguccuAaug...	1	1	JCK-3
...aucacuaauacugcaguccucaug...	23	0	JCK-2
...aucacuaauacugUaguccucaug...	1	1	JCK-2
...aucacuaauacugcaguccucauA...	1	1	JCK-3
...aucacuaauacugcaguccucaug...	2	0	LT-1
...aucacuaauacugcUguccucaug...	1	1	LT-3
...aucacuaauacugUaguccucaug...	1	1	JCK-3
...aucacuaauacugcagucUucaug...	1	1	LT-1
...aucacuaauacugcaguccucaug...	11	0	JCK-3
...aucacuaauacugcaguccucauA...	1	1	JCK-2
...aucacuaauacugcaguccucaug...	10	0	JCK-1
...aucaAuaauacugcaguccucaug...	3	1	JCK-2
...aucacuaauacugcaguccucaugc...	2	0	LT-1
...Acacuaauacugcaguccuca...	2	1	LT-2
...ucacuaauacugcaguccucaugc...	14	0	JCK-1
...ucacuaauacugcaguccucaugc...	11	0	LT-2
...ucacuaauacugcaguccucaugc...	28	0	JCK-2
...ucacuaauacugcaguccucaugc...	29	0	LT-3
...ucacuaauacugcaguccucaugc...	35	0	LT-1
...ucaAuaauacugcaguccucaugc...	1	1	JCK-2
...ucacuaauacugcaguccucaugc...	18	0	JCK-3
...ucacuaauacugcaAuccucaugc...	2	1	JCK-2
...ucaUuaauacugcaguccucaugc...	1	1	LT-3
...ucacuaauacugcaguAcucaugc...	9	1	LCK-3
...ucacuaauacugcaguAcucaugc...	106	1	JCK-2
...ucacuaauacugcaguAcucaugc...	25	1	LT-1
...ucacuaauacugcaguAcucaugc...	43	1	JCK-1
...ucaUuaauacugcaguccucaugc...	1	1	LT-2
...ucacuaauacugcaAuccucaugc...	2	1	JCK-3
...ucaUuaauacugcaguccucaugc...	1	1	JCK-3
...Acacuaauacugcaguccucaugc...	17	1	LT-1
...ucacuaauacugcaguAcucaugc...	9	1	JT-1
...uUacuaauacugcaguccucaugc...	2	1	JCK-2
...ucacuaauacugcaguGcucaugc...	3	1	LT-2
...ucacuaauacugcaguccucaugc...	15	0	LCK-3
...ucaAuaauacugcaguccucaugc...	1	1	JCK-3
...uUacuaauacugcaguccucaugc...	1	1	LT-1
...uUacuaauacugcaguccucaugc...	4	1	JCK-3
...ucaUuaauacugcaguccucaugc...	2	1	JCK-1
...ucacuaauacugcaguGcucaugc...	3	1	LT-3
...ucacuaauacugcaguGcucaugc...	16	1	LCK-3
...ucacuaauacugcaAuccucaugc...	1	1	JCK-1
...ucacAaauacugcaguccucaugc...	2	1	LT-2
...ucacAaauacugcaguccucaugc...	2	1	JCK-1
...ucacuaauacugcaguAcucaugc...	19	1	LT-2
...ucacuaauacugcaguGcucaugc...	2	1	JCK-1
...ucacuaauacugcaguGcucaugc...	3	1	LT-1
...Ccacuaauacugcaguccucaugc...	3	1	JCK-2
...ucacAaauacugcaguccucaugc...	5	1	JCK-2
...ucacuaauacugcCguccucaugc...	1	1	LT-1
...ucacuaauacugcaguAcucaugc...	31	1	LT-3
...ucaUuaauacugcaguccucaugc...	2	1	LT-1
...ucacuaauacugcaguGcucaugc...	2	1	JCK-2
...ucacuaauacugcaguAcucaugc...	64	1	JCK-3
...Ccacuaauacugcaguccucaugc...	1	1	LT-3
...ucacAaauacugcaguccucaugc...	6	1	LT-3
...ucacuaauacugcaguGcucaugc...	1	1	JCK-3

Star | Mature

augugaucauaguaaauaagggguucagauacauugugaucauaguaaauuagguuuuagauguauauauauauauauauauauauauauauauaucaguccguuuauagugcggauggucuucauacagaccacaguauuggugacgguuuuuaauaguauuggugacgauuuucuaagaaaccgucguuaauacuaugaaaaaccaucacuaauacugcaguccucaugcggaccguauucacuauagaau

ucacAaauacugcaguccucaugc	2	1	JCK-3
uUacuaauacugcaguccucaugc	1	1	JCK-1
Acacuaauacugcaguccucaugc	9	1	LT-3
cacuaauacugcaguccuc	1	0	JCK-3
cacuaauacugcaguccuca	2	0	JCK-2
cacuaauacugcagucUuca	5	1	LT-2
cacuaauacugcaguGcuca	4	1	LT-2
cacuaauacugcaAuccucaug	1	1	LT-1
cacuaauacugcaguAcucaugc	1	1	JCK-1
caUuaauacugcaguccucaugc	7	1	LT-2
cacuaauacugcaguGcucaugc	3	1	LT-3
cacuaauacugcaguccucaugc	35	0	LT-3
cacuaauacugcaguAcucaugc	5	1	LT-1
cacuaauacugcaguccucaugc	16	0	LCK-3
cacuaauacugcaguAcucaugc	2	1	LT-2
caUuaauacugcaguccucaugc	2	1	LT-1
cacuaauacugcaguccucaugc	45	0	LT-1
cacAaauacugcaguccucaugc	2	1	JCK-3
cacAaauacugcaguccucaugc	1	1	JCK-2
cacuaauCcugcaguccucaugc	1	1	LT-1
caUuaauacugcaguccucaugc	4	1	LT-3
Uacuaauacugcaguccucaugc	1	1	JCK-3
cacuaauacugcaguAcucaugc	2	1	LCK-3
caUuaauacugcaguccucaugc	3	1	JCK-2
cacuaauacugcaguccucaugc	11	0	JCK-1
cacuaauacugcaguGcucaugc	2	1	LT-1
cacuaauacugcaguAcucaugc	2	1	LT-3
cacuaauacugcUguccucaugc	2	1	JCK-1
cacuaauacugcaguccucaugc	19	0	JCK-2
cacuaauacugcUguccucaugc	1	1	JCK-2
cacuaauacugcaguccucaugc	9	0	JCK-3
cacuaauacugcaguccucaugc	46	0	LT-2
cacAaauacugcaguccucaugc	7	1	LT-1
cacAaauacugcaguccucaugc	2	1	LT-3
caAuaauacugcaguccucaugc	2	1	JCK-2
cacuaauacugcaguAcucaugc	6	1	JCK-2
cacuaauacugcaguAcucaugc	2	1	JCK-3
caUuaauacugcaguccucaugc	7	1	JCK-3
cacuaauacugcaguGcucaugc	2	1	LCK-3
cacuaauacugcaguAcucaugcg	1	1	LT-1
cacuaauacugcaguccucaugcg	2	0	LT-1
cacuaauacugcaguccucaugUg	1	1	LT-3
cacAaauacugcaguccucaugcg	3	1	JCK-3
cacuaauacugcaguccucaugcg	4	0	JCK-1
cacuaauacugcaguAcucaugcg	2	1	LT-3
cacuaauacugcaguAcucaugcg	3	1	JCK-2
caUuaauacugcaguccucaugcg	1	1	LT-2
cacuaauacugcaguccucauNcg	1	1	JCK-1
cacuaauacugcaguccucaugcg	4	0	LT-3
cacuaauacugcaguccucaugcg	3	0	JCK-3
cacuaauacugcaguccucaugcA	1	1	LT-3
cacuaauacugcaguccucaugcg	2	0	JCK-2
cacuaauacugcaguAcucaugcg	2	1	JCK-3
cacuaauacugcaguGcucaugcg	1	1	LT-1
cacuaauacugcaguAcucaugcg	3	1	JCK-1
acAaauacugcaguccucaug	1	1	JCK-3
acuaauacugcaguccucauC	1	1	JCK-3
acuaauacugcagucUucaug	1	1	LT-3
acuaauacugcaguccucauC	1	1	JCK-1
acuaauacugcaguGcucaug	1	1	JCK-2
acuaauacugcaguccucaug	1	0	JCK-3
acuaauacugcaguccucaug	3	0	JCK-2
acuaauacugcaguccucaug	1	0	LT-1
acAaauacugcaguccucaug	1	1	JCK-2
acuaauacugcaguccucaug	2	0	JCK-1
aUuaauacugcaguccucaug	1	1	LT-3
acuaauacugcaguccucaugc	4	0	JT-2
acAaauacugcaguccucaugc	1	1	LT-3
acuaauacugcaguccucaugc	2	0	LT-3
acuaauacugcaguGcucaugc	1	1	LT-1

Star Mature

augugaucauaguaaauaaggguucagauacauugugaucauaguaaauuagguuuuagauguauauauauauauauauauauauauauauauaucaguccguuuauagugcggauggucuucauacagaccacaguauuggugacgguuuuuaauaguauuggugacgauuuucuaagaaaccgucguuaauacuaugaaaaaccaucacuaauacugcaguccucaugcggaccguauucacuauagaau

acuaauacugcaguccucaugc	4	0	LT-2
acuaauacugcaguccucaugc	5	0	LT-1
acuaauacugcaguccucaugc	4	0	JT-3
acuaauacugcaguGcucaugc	1	1	LT-3
acuaauacugcaguccucaugc	5	0	JCK-2
aUuaauacugcaguccucaugc	2	1	LT-2
acuaauacugcUguccucaugcg	1	1	LT-1
cuaauacugcagucUucaug	1	1	LT-1
cuaauacugcaguGcucaug	2	1	LT-1
cuaauacugcagucUucaug	1	1	JCK-2
cuaauacugcaguccucaug	2	0	LT-2
cuaauacugcaguccucaug	1	0	JCK-2
cuaauacugcaguGcucaug	2	1	LT-2
cuaauacugcaguccucaug	1	0	LT-1
cuaauacugcaguGcucaugc	3	1	JCK-3
cuaauacugcaguGcucaugc	5	1	JCK-2
Uuaauacugcaguccucaugc	3	1	JCK-2
cuaauacugcaguccucaugc	38	0	LT-3
cuaauacugcaguccucaugc	25	0	LT-2
Uuaauacugcaguccucaugc	1	1	JCK-1
Uuaauacugcaguccucaugc	5	1	LT-1
cuaauacugcaguccucaugc	49	0	LCK-3
cuaauCcugcaguccucaugc	1	1	JCK-3
cuaauacugcaguGcucaugc	3	1	LCK-3
cuaauacugcaguccucaugc	2	0	JT-2
cuaauacugcaguGcucaugc	7	1	LT-3
cCaauacugcaguccucaugc	1	1	LCK-2
cuaauacugcaguccucaugc	6	0	JT-3
cAaauacugcaguccucaugc	21	1	LCK-1
cuaauacugcaguGcucaugc	6	1	LT-1
cuaauacugcaguccucaugc	17	0	JCK-3
Uuaauacugcaguccucaugc	10	1	LCK-3
Auaauacugcaguccucaugc	1	1	LT-3
cuaauacugcaguccucaugc	19	0	JCK-1
cAaauacugcaguccucaugc	28	1	LCK-2
Uuaauacugcaguccucaugc	6	1	LT-2
cuaauacugcaguGcucaugc	5	1	JCK-1
cuaauacugcaguccucaugc	21	0	JCK-2
cAaauacugcaguccucaugc	1	1	JCK-1
cAaauacugcaguccucaugc	2	1	LT-3
cuaauacugcUguccucaugc	1	1	LT-3
cuNauacugcaguccucaugc	1	1	LT-2
cuaauacugcaguccucaugc	34	0	LT-1
cAaauacugcaguccucaugc	1	1	JCK-2
Uuaauacugcaguccucaugc	1	1	LT-3
cuaauacugcaguGcucaugc	13	1	LT-2
cuaauacugcaguccucaugU	1	1	LT-3
cuaauacugcaguccucaugUg	1	1	LT-2
cuaauacugcaguccucaugcA	2	1	LT-2
cuaauacugcaguccucaugcg	6	0	LCK-3
cuaauacugcaguccucaugcgU	1	1	LT-3
Aaauacugcaguccucaugc	1	1	JCK-3
uaauacugcaguccucaugc	3	0	LT-1
uaauacugcaguccucaugc	2	0	LT-3
uaauacugcaguccucaugc	6	0	JCK-1
uaauacugcaguccucaugc	12	0	LCK-3
uaauacugcaguccucaugc	5	0	JCK-2
uaauacugcaguccucaugcg	6	0	JT-2
uaauacugcaguccucaugcg	5	0	LCK-3
uaauacugcaguccucaugcg	9	0	JT-3
uaauacugcUguccucaugcg	1	1	LT-1
uaauacugcaguccucaugcg	1	0	LT-1
uaauacugcaguGcucaugcg	2	1	LT-1
uaauacugcaguccucaugcgAa	6	1	LT-2
aauacugcaguGcucaugc	3	1	LT-3
aauacugcaguGcucaugc	7	1	JT-3
aauacugcaguccucaugc	5	0	LT-1
aauacugcaguccucaugc	3	0	JCK-2
aauacugcaguGcucaugc	5	1	JT-2
aauacugcaguccucaugc	3	0	JCK-1

Star **Mature**

augugaucauaguaaauaaggguucagauacauugugaucauaguaaauuagguuuuagauguauauauauauauauauauauauauauaucaguccguuuauagugcggauggucuucauacagaccacaguauuggugacgguuuuuaauaguauuggugacgauuuucuaagaaaccgucguuaauacuaugaaaaaccaucacuaauacugcaguccucaugcggaccguauucacuauagaau

aauacugcaguGcucaugc	4	1	LCK-3
aauacugcaguccucaugc	3	0	LCK-3
aauacugcaguccucaugc	6	0	LT-3
aauacugcaguccucaugc	1	0	JCK-3
aauacugcUguccucaugc	1	1	JCK-3
aauacugcaguccucaugc	7	0	LT-2
aauacugcaguccucaugUgga	2	1	LT-3
aauacugcaguccucaugcgAac	2	1	LT-3
aauacugcaguGcucaugcggacc	1	1	JCK-2
aauacugcaguGcucaugcggacc	1	1	JCK-3
aauacugcaguGcucaugcggacc	1	1	JCK-1
aauacugcaguGcucaugcggacc	8	1	LCK-3
auacugcaguccucaugc	1	0	LT-1
auacugcaguccucaugc	1	0	JCK-2
auacugcaguccucaugc	3	0	LCK-3
auacugcaguccucaugcggacc	2	0	LT-2
uacugcaguccucaugcgAacc	1	1	LT-1
uccucaugcggacUguauu	2	1	JCK-3
cucaugcggaccguaCucacu	1	1	LT-1
cucaugcggaccguCuucacu	1	1	JCK-1
cucaugcggaccguCuucacu	1	1	JCK-3
ucaugcggaccguCuucacu	1	1	LT-3
caugcggaccguUuucacu	1	1	JCK-2
caugcggaccguUuucacu	1	1	JCK-3
cauAcggaccguauucacuau	1	1	JCK-3
augcggaccguaCucacuaua	1	1	LT-3
